# Global DNA demethylation as an epigenetic marker of human brain metastases

**DOI:** 10.1042/BSR20180731

**Published:** 2018-10-23

**Authors:** Anna-Maria Barciszewska

**Affiliations:** 1 Intraoperative Imaging Unit, Chair and Clinic of Neurosurgery and Neurotraumatology, Karol Marcinkowski University of Medical Sciences, Przybyszewskiego 49, 60-355 Poznan, Poland; 2 Department of Neurosurgery and Neurotraumatology, Heliodor Swiecicki Clinical Hospital, Przybyszewskiego 49, 60-355 Poznan, Poland

**Keywords:** biomarkers, epigenetics, methylation, 5-methylcytosine, metastasis, molecular diagnostics

## Abstract

Brain metastases are the most common intracranial tumors in adults. They usually originate from: lung, breast, renal cell and gastrointestinal cancers, as well as melanoma. Prognosis for brain metastases is still poor and classical treatment combining surgery and radiation therapy should be strongly supported with molecular approaches. However, their successful application depends on a deep understanding of not only genetic, but also epigenetic background of the disease. That will result in an earlier and more precise diagnosis, successful treatment, as well as individualized estimation of clinical outcomes and prognosis. It has already been shown that the epigenetic machinery plays a crucial role in cancer biology, development, and progression. Therefore, we decided to look for metastasis through changes in the most studied epigenetic mark, 5-methylcytosine (m^5^C) in DNA. We performed global analysis of the m^5^C contents in DNA isolated from the brain metastatic tumor tissue and peripheral blood samples of the same patients, using thin layer chromatography separation of radioactively labeled nucleotides. We found that the m^5^C level in DNA from brain metastases: changes in the broad range, overlaps with that of blood, and negatively correlates with the increasing tumor grade. Because the amount of m^5^C in tumor tissue and blood is almost identical, the genomic DNA methylation can be a useful marker for brain metastases detection and differentiation. Our research creates a scope for future studies on epigenetic mechanisms in neuro-oncology and can lead to development of new diagnostic methods in clinical practice.

## Introduction

The incidence of brain metastases is ten times higher than that of primary malignant brain tumors. Nearly half of the patients with systemic cancer will develop central nervous system (CNS) metastases during the course of their disease and with increased surveillance and improved systemic control, the incidence is likely to grow [1]. A diagnosis of brain metastasis carries a dismal prognosis, especially in older patients, with poor performance status (Karnofsky Performance Score, KPS) below 70, where median overall survival (OS) is 2.3 months. For younger patients (age <65) with good performance status (KPS >70) OS is approximately 7.1 months [2]. Early detection of brain metastases is a result of more precise and innovative neuroimaging modalities, as well as improvements in oncology treatments that are leading to longer patient’s survival [3], but also to an increasing incidence of brain metastases [4,5]. In adults, metastases to the brain most commonly arise from primary tumors of the lung (50–60%), and breast (15–20%), followed by skin (melanoma), and gastrointestinal tract [6,7]. Melanoma, testicular and renal carcinomas have the greatest propensity to metastasize to the brain, but their relative rarity explains the low incidence of these neoplasms in a large series of patients with brain metastases [8–10].

Unique interactions between the brain’s micro-environment, blood–brain barrier, and tumor cells are hypothesized to promote distinct molecular features in CNS metastases [11]. The mechanisms governing the cancer development are thought to be the result of not only genetic defects, but also epigenetic modifications. Therefore, epigenetics has become a very attractive and increasingly investigated field of research in order to find new methods for prevention, diagnosis, and treatment of neoplasms [12,13].

Methylation of DNA cytosine residue at the carbon 5 position (5-methylcytosine, m^5^C) is one of the most recognized epigenetic mechanism controlling gene expression, which can dynamically respond to external and internal factors [14–16]. It is often found in CpG islands. It is assumed that ca. 5% of all cytosine residues, i.e. 1% of the nucleic bases, in mammalian genomes are methylated [17]. Although DNA methylation has been regarded as a stable epigenetic mark, various studies revealed that this modification is not static at all [14]. Loss of DNA methylation (DNA hypomethylation) occurs through active, passive, or random modification mechanisms [18,19]. In the CNS, epigenetic mechanisms serve as main regulators of homeostasis and plasticity development, which are sensitive to local and global, environmental as well as intrinsic factors [20].

It is generally accepted that cancer development and progression is linked to the disruption of red-ox balance of the cell [21] and indeed induced by enhanced reactive oxygen species (ROS) generation, their accumulation, and down-regulation of antioxidant enzymes [22–24]. ROS cause damage to DNA and other cell components, induce epigenetic alterations, interact with oncogenes or tumor suppressor genes, and finally modulate immunological responses [15,25].

The most reactive and dangerous ROS is the hydroxyl radical (·OH). Because of its short lifetime (10^−9^ s) it causes a wide range of DNA lesions including nucleic acid base modifications, deoxyribose degradation, deletions, and strands breakage. Hydroxyl radical oxidation of m^5^C leads to its demethylation and deamination, as well as 5-methyluridine formation. It results in the global (genomic) hypomethylation of cellular DNA. Therefore DNA methylation (m^5^C status) is a sensitive marker of the neoplasm formation as an effect of the oxidative stress, ROS formation and damage reactions, very characteristic for cancer [26,27].

Measurements of m^5^C level in DNA can be done either by analyzing the pattern of methylated target sequences along individual DNA molecules or as an average methylation level at a single genomic locus across many DNA molecules [28,29].

To investigate whether hypomethylation plays a role in brain metastasis development, we have analyzed the global DNA methylation level in human brain tumor tissues and peripheral blood samples from patients with brain metastasis from different sites of origin. For estimation of m^5^C content we used a very sensitive two-dimensional thin layer chromatography (TLC) technique of separation of radioactively labeled nucleotides ([γ^32^P] postlabeling method) [30,31]. We observed a reversed relationship of global m^5^C content in DNA of tumor tissues to their grade. The association with the site of origin was also found. Moreover, the correlation between global DNA methylation in tumor tissues and peripheral blood samples from the same patients was observed. Through a detailed analysis of the global DNA methylation in brain metastasis patients we propose an epigenetic method of brain metastasis characterization with potential practical application in clinical diagnostic and treatment monitoring.

## Materials and methods

### Ethics approval and consent to participate

Blood and tissue molecular analysis was approved by the Bioethical Committee of Karol Marcinkowski University of Medical Sciences, Poznan (896/9; 838/12). All participants provided written consent and indicated willingness to donate their blood and tissue samples for research.

### Collection of tumor tissue and peripheral blood samples

The brain metastasis tissue samples were collected from 139 patients who underwent brain tumor resection at the Department of Neurosurgery and Neurotraumatology of the University of Medical Sciences in Poznan between 2004 and 2012. In 45 of those patients samples of peripheral blood were also collected preoperatively. The peripheral blood samples were also taken from 41 generally healthy individuals comprising the control group. Tumor tissues and peripheral blood samples were immediately frozen and stored at −80°C.

Patient’s demographic data were obtained from the patient’s medical records. Brain tumor tissues were routinely and neuropathologically evaluated to determine histological types and grades.

### DNA isolation from tumor tissue samples

Genomic DNA was extracted from frozen tumor tissue samples with a commercially available kit (A&A Biotechnology). The samples were incubated with proteinase K and then with RNase A. The supernatant, obtained after centrifugation was applied to mini column. DNA was eluted with Tris-buffer pH 8.5 and stored at −20°C for further analysis. DNA purity was checked by measuring the UV absorbance at 260 and 280 nm. The A_260_/A_280_ ratio was 2.0–2.1.

### DNA isolation from peripheral blood samples

DNA from 7.5 ml of human blood was isolated by lysis with 30 ml of cold (4°C) buffer of 155 mM NH_4_Cl, 10 mM KHCO_3_, and 0.1 mM Na_2_EDTA pH 7.4 for 30 min, then centrifuged at 3000 rpm for 10 min in 4°C. The pellets were resuspended in 10 ml of above mentioned buffer and centrifuged again. The cell lysate was resuspended in 5 ml of buffer containing 75 mM NaCl, 1 mM Na_2_EDTA, pH 8.0 and digested with 25 μl protease K solution (10 μg/μl), and 250 μl 20% SDS for 16 h at 55°C. Finally 1500 μl of 5 M NaCl was added and tube-shaked vigorously for 15 min, then centrifuged at 4000 rpm for 15 min in room temperature. The DNA was precipitated with two volumes of cold ethanol, removed with pipette and dissolved in 100 μl of distilled water. The purity of DNA preparations was checked by measuring the UV absorbance at 260 and 280 nm. The A_260_/A_280_ ratio was 2.0–2.1.

### DNA hydrolysis, labeling, and TLC chromatography

**Figure 1:**
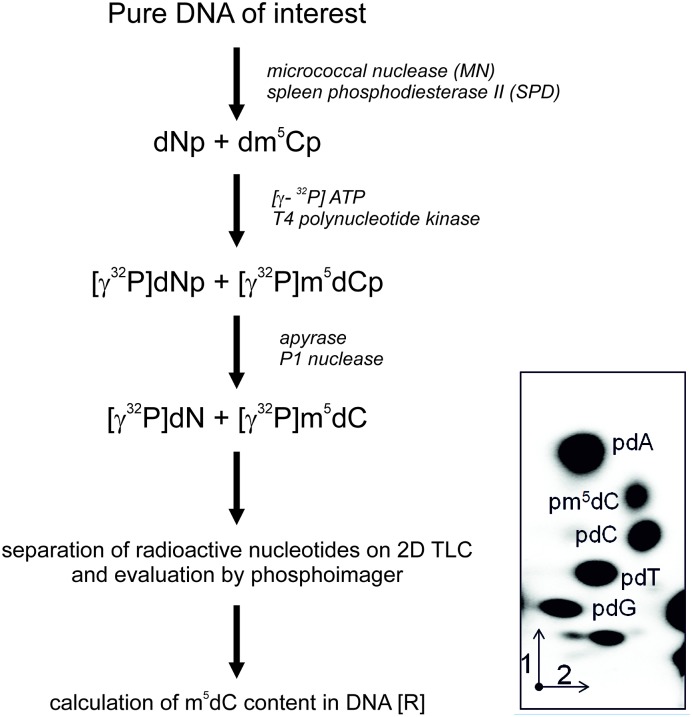
Flow chart of m^5^C analysis in DNA hydrolyzed to 3’-mononucleotides (Np, A - adenosine, G - guanosine, C - cytidine, T - thymidine). They are further labeled with [γ-^32^P] ATP, dephosphorylated of 3’ phosphate and separated with TLC in two dimensions (1 and 2). The chromatogram was evaluated with phosphoimager and the spots’ intensities were measured. These values were used for the calculation of the R coefficient according to the given equation [30,31].

One microgram of dried DNA was dissolved in a succinate buffer (pH 6.0) containing 10 mM CaCl_2_ and digested with 0.001 units of spleen phosphodiesterase II and 0.02 units of micrococcal nuclease in 3.5 μl total volume for 5 h at 37°C. Also, 0.17 μg of DNA digest was labeled with 1 μCi [γ-^32^P] ATP (6000 Ci/mM; Hartmann Analytic GmbH) and 1.5 units of T4 polynucleotide kinase in 3 μl of 10 mM bicine-NaOH pH 9.7 buffer containing 10 mM MgCl_2_, 10 mM DTT, and 1 mM spermidine. After 30 min at 37°C, 3 μl of apyrase (10 units/ml) in the same buffer were added and incubated for another 30 min. The 3′ nucleotide phosphates were cleaved off with 0.2 μg RNase P1 in 500 mM ammonium acetate buffer, pH 4.5. Identification of [γ-^32^P]m^5^dC was performed by a two-dimensional thin-layer chromatography on cellulose plates (Merck, Germany) using solvent system: isobutyric acid:NH_4_OH:H_2_O (66:1:17 v/v) in the first dimension and 0.2 M sodium phosphate (pH 6.8)-ammonium sulfate-*n*-propyl alcohol (100 ml/60 g/1.5 ml) in the second dimension. Radioactive spot analysis was done with the Phosphoimager Typhoon Screen (Pharmacia, Sweden) and ImageQuant Software (GE Healthcare, U.S.A.). The analysis was repeated three times for each probe and results were evaluated with the statistic software. For calculation, the amount of radioactive material corresponding to m^5^dC, dC (cytosine) and dT (thymine) was used. The global DNA methylation was calculated as R = (m^5^dC/(m^5^dC+dC+dT)) × 100 (Figure 1), because cytosine and thymine are formed in the m^5^dC oxidation process [32].

### Statistical analysis

Statistical analysis (descriptive statistics, ANOVA test, correlation) was performed with EXCEL software.

## Results

### Patients’ characteristics

The analyzed cohort consisted of 139 individuals diagnosed with brain metastasis, aged from 32 to 77 years. The largest subgroup consisted of patients within the age range of 51–60 years. The median age of patients at the time of tumor diagnosis was 57.4 ± 7.8 years. There were 82 (59.0%) males and 57 (41.0%) females.

The most common site of origin in analyzed cohort was lung cancer (70.5% of cases, of which 88.8% were non-small cell lung cancer [NSCLC]), followed by colon cancer and skin melanoma (both 5.7% of cases) and breast cancer (5.0%). Single cases represented other primary tumor sites (Table 1). The histological types and grades (from G1 – highly differentiated, least malignant to G3 – low differentiated, most malignant) as well as numeric data from DNA methylation analysis are shown in Table 1.

**Table 1:** The list of patients with brain metastases evaluated in the present study

Case	Histological type of brain metastasis	Grade	Site of origin	Sex	Age	R tissue	SD R tissue	R blood	SD R blood
1.	Adenocarcinoma	G3	Lung	F	54	0.12	0.01	0.36	0.02
2.	Melanoma	–	Skin	F	39	0.18	0.01	–	–
3.	Small cell carcinoma	G3	Lung	F	66	0.26	0.04	–	–
4.	Adenocarcinoma	G3	Lung	F	62	0.27	0.07	0.33	0.06
5.	Ductal carcinoma	–	Breast	F	67	0.28	0.03	–	–
6.	Planoepithelial carcinoma	G3	Lung	M	69	0.28	0.03	0.48	0.02
7.	Transitional carcinoma	–	Bladder	M	62	0.29	0.08	–	–
8.	Anaplastic small cell carcinoma	G3	Lung	M	67	0.29	0.08	–	–
9.	Tubular adenocarcinoma	G3	Colon	M	51	0.31	0.06	0.24	0.02
10.	Adenocarcinoma	–	Lung	F	49	0.32	0.02	0.49	0.02
11.	Papillary carcinoma	–	Thyroid	F	54	0.35	0.05	0.49	0.05
12.	Adenocarcinoma	G3	Lung	F	62	0.36	0.12	–	–
13.	Adenocarcinoma	–	Lung	F	46	0.38	0.07	–	–
14.	Papillary adenocarcinoma	–	Endometrium	F	60	0.38	0.04	–	–
15.	Adenocarcinoma	G3	Lung	M	55	0.38	0.04	0.36	0.04
16.	Adenocarcinoma	–	Lung	M	50	0.41	0.05	–	–
17.	Papillary adenocarcinoma	G3	Lung	M	52	0.43	0.03	–	–
18.	Melanoma	–	Skin	F	32	0.45	0.11	–	–
19.	Adenocarcinoma	–	Endometrium	F	63	0.45	0.09	–	–
20.	Papillary adenocarcinoma	–	Lung	F	68	0.45	0.03	–	–
21.	Adenocarcinoma	G3	Oesophagus	M	63	0.45	0.05	–	–
22.	Small cell carcinoma	G3	Lung	M	58	0.46	0.05	0.52	0.08
23.	Adenocarcinoma	G3	Lung	M	71	0.46	0.02	0.46	0.02
24.	Papillary adenocarcinoma	G2	Lung	F	64	0.47	0.02	0.43	0.02
25.	Papillary adenocarcinoma	–	Lung	M	66	0.48	0.04	–	–
26.	Planoepithelial carcinoma	G3	Lung	M	72	0.48	0.09	–	–
27.	Solid carcinoma	–	Lung	M	73	0.48	0.03	–	–
28.	Adenocarcinoma	G3	Lung	F	65	0.49	0.07	–	–
29.	Papillary adenocarcinoma	–	Lung	F	68	0.49	0.05	–	–
30.	Adenocarcinoma	–	Lung	M	56	0.49	0.07	–	–
31.	Clear cell renal cell carcinoma	–	Kidney	M	56	0.49	0.07	–	–
32.	Planopithelial carcinoma	G3	Lung	M	64	0.49	0.07	1.12	0.05
33.	Small cell carcinoma	G3	Lung	M	65	0.49	0.07	0.56	0.04
34.	Adenocarcinoma	G3	Lung	F	59	0.50	0.10	–	–
35.	Solid carcinoma	–	Lung	F	62	0.50	0.08	–	–
36.	Adenocarcinoma	–	Lung	M	50	0.50	0.08	–	–
37.	Papillary adenocarcinoma	–	Colon	M	60	0.50	0.10	0.69	0.04
38.	Anaplastic small cell carcinoma	G3	Lung	F	48	0.52	0.10	–	–
39.	Anaplastic small cell carcinoma	G3	Lung	F	66	0.52	0.05	–	–
40.	Adenocarcinoma	–	Lung	M	48	0.52	0.08	–	–
41.	Adenocarcinoma	–	Lung	M	49	0.52	0.08	–	–
42.	Melanoma	–	Skin	M	51	0.52	0.07	–	–
43.	Papillary adenocarcinoma	–	Lung	M	56	0.52	0.07	–	–
44.	Adenocarcinoma	G2	Lung	M	61	0.52	0.01	0.43	0.18
45.	Solid carcinoma	–	Lung	F	52	0.53	0.05	0.52	0.03
46.	Planopithelial carcinoma	G3	Lung	F	54	0.53	0.07	–	–
47.	Solid carcinoma	–	Lung	F	66	0.53	0.04	–	–
48.	Papillary adenocarcinoma	–	Lung	F	44	0.54	0.04	–	–
49.	Ductal carcinoma	–	Breast	F	50	0.54	0.09	–	–
50.	Solid carcinoma, poorly differentiated	G3	Bladder	F	52	0.54	0.06	–	–
51.	Papillary adenocarcinoma	–	Lung	F	59	0.54	0.07	–	–
52.	Adenocarcinoma	–	Lung	F	55	0.55	0.07	–	–
53.	Adenocarcinoma	G2	Colon	F	56	0.55	0.05	–	–
54.	Amelanotic melanoma	–	Skin	M	44	0.55	0.07	–	–
55.	Solid carcinoma	–	Lung	M	65	0.55	0.05	–	–
56.	Adenocarcinoma	–	Lung	F	48	0.56	0.09	–	–
57.	Solid carcinoma	–	Lung	M	50	0.56	0.08	–	–
58.	Anaplastic small cell carcinoma	G3	Lung	M	64	0.56	0.04	0.68	0.04
59.	Melanoma	–	Skin	F	55	0.57	0.05	0.72	0.06
60.	Melanoma	–	Skin	M	54	0.57	0.05	–	–
61.	Adenocarcinoma	–	Lung	M	57	0.57	0.07	–	–
62.	Papillary carcinoma	–	Lung	M	62	0.57	0.05	–	–
63.	Adenocarcinoma	–	Lung	M	70	0.57	0.05	–	–
64.	Adenocarcinoma	–	Lung	F	47	0.58	0.04	–	–
65.	Solid carcinoma	–	Lung	F	48	0.58	0.08	–	–
66.	Papillary adenocarcinoma	G2	Lung	F	68	0.58	0.08	–	–
67.	Adenocarcinoma	–	Lung	M	49	0.58	0.03	0.97	0.05
68.	Adenocarcinoma	G3	Lung	M	58	0.58	0.05	–	–
69.	Adenocarcinoma	–	Lung	M	58	0.58	0.03	–	
70.	Anaplastic small cell carcinoma	G3	Lung	F	40	0.59	0.06	–	–
71.	Adenocarcinoma	G2	Lung	F	48	0.59	0.05	0.58	0.08
72.	Papillary adenocarcinoma	G2	Lung	M	60	0.59	0.06	0.43	0.07
73.	Papillary adenocarcinoma	–	Lung	M	50	0.60	0.08	–	–
74.	Solid carcinoma, poorly differentiated	G3	Lung	M	62	0.60	0.06	0.36	0.02
75.	Solid carcinoma	–	Lung	M	72	0.60	0.03	0.33	0.02
76.	Papillary adenocarcinoma	–	Breast	F	55	0.61	0.12	–	–
77.	Clear cell adenocarcinoma	–	Lung	M	58	0.61	0.07	–	–
78.	Planoepithelial carcinoma, akeratotic	G3	Lung	M	59	0.61	0.06	0.78	0.03
79.	Solid carcinoma	–	Lung	F	48	0.62	0.07	–	–
80.	Solid carcinoma	–	Lung	M	64	0.63	0.07	–	–
81.	Adenocarcinoma	–	Prostate	M	46	0.64	0.03	–	–
82.	Solid carcinoma, poorly differentiated	G3	Lung	M	56	0.64	0.09	–	–
83.	Papillary adenocarcinoma	G2	Lung	M	57	0.64	0.04	0.65	0.06
84.	Solid carcinoma	–	Lung	M	46	0.66	0.13	–	–
85.	Planoepithelial carcinoma, akeratotic	G2	Lung	M	66	0.66	0.15	–	–
86.	Adenocarcinoma	G3	Lung	M	66	0.66	0.09	0.53	0.02
87.	Sarcoma	–	Endometrium	F	62	0.67	0.08	–	–
88.	Papillary adenocarcinoma	–	Lung	M	65	0.67	0.16	–	–
89.	Solid carcinoma, poorly differentiated	G3	Lung	M	65	0.69	0.07	–	–
90.	Adenocarcinoma	–	Lung	M	73	0.69	0.05	0.67	0.06
91.	Papillary adenocarcinoma	G2	Breast	F	51	0.70	0.10	0.98	0.05
92.	Adenocarcinoma	–	Lung	F	49	0.71	0.02	–	–
93.	Embryonal carcinoma	–	Testis	M	52	0.71	0.06	–	–
94.	Clear cell adenocarcinoma	–	Lung	M	56	0.71	0.02	–	–
95.	Large cell carcinoma	G3	Lung	F	56	0.72	0.06	–	–
96.	Adenocarcinoma	–	Lung	M	51	0.72	0.04	–	–
97.	Adenocarcinoma	–	Colon	M	54	0.72	0.01	–	–
98.	Large cell carcinoma	G3	Lung	M	54	0.72	0.01	0.94	0.02
99.	Large cell carcinoma	G3	Lung	M	65	0.73	0.06	–	–
100.	Melanoma	–	Skin	M	77	0.73	0.06	0.98	0.06
101.	Embryonal carcinoma	–	Testis	M	46	0.74	0.09	–	–
102.	Solid carcinoma, poorly differentiated	G3	Lung	M	51	0.75	0.05	0.54	0.01
103.	Melanoma	–	Skin	M	63	0.75	0.09	–	–
104.	Adenocarcinoma	G3	Lung	M	63	0.76	0.12	–	–
105.	Papillary adenocarcinoma	G2	Breast	F	68	0.77	0.08	0.68	0.04
106.	Papillary adenocarcinoma	G2	Lung	F	60	0.78	0.09	–	–
107.	Adenocarcinoma	–	Breast	M	57	0.78	0.05	–	–
108.	Adenocarcinoma	–	Lung	M	62	0.79	0.07	–	–
109.	Adenocarcinoma	–	Lung	F	57	0.82	0.11	–	–
110.	Planoepitheliale carcinoma	G3	Lung	M	49	0.82	0.09	–	–
111.	Anaplastic small cell carcinoma	G3	Lung	M	50	0.82	0.03	0.88	0.02
112.	Tubular papillary adenocarcinoma	G2	Colon	M	53	0.82	0.09	–	–
113.	Planoepithelial carcinoma	–	Tongue	M	55	0.82	0.09	–	–
114.	Large cell carcinoma	–	Lung	M	61	0.82	0.06	0.61	0.07
115.	Adenocarcinoma	–	Breast	F	53	0.84	0.04	–	–
116.	Papillary adenocarcinoma	G2	Lung	M	64	0.84	0.09	0.95	0.04
117.	Papillary adenocarcinoma	G2	Lung	F	56	0.85	0.02	0.67	0.06
118.	Adenocarcinoma	G3	Lung	M	52	0.85	0.04	0.76	0.01
119.	Tubular adenocarcinoma	G2	Colon	M	49	0.86	0.07	0.70	0.05
120.	Papillary adenocarcinoma	G2	Lung	M	55	0.86	0.07	–	–
121.	Papillary adenocarcinoma	G2	Lung	M	58	0.87	0.06	0.86	0.19
122.	Adenocarcinoma	G1	Lung	F	54	0.92	0.04	–	–
123.	Papillary adenocarcinoma	–	Lung	M	67	0.93	0.10	–	–
124.	Papillary adenocarcinoma	G1	Lung	M	69	0.93	0.04	0.96	0.04
125.	Small cell carcinoma	–	Lung	M	57	0.96	0.04	–	–
126.	Papillary adenocarcinoma	–	Lung	F	58	0.97	0.03	1.19	0.07–
127.	Papillary adenocarcinoma	G3	Lung	M	60	0.98	0.03	0.96	0.01
128.	Papillary adenocarcinoma	–	Ovary	F	61	0.99	0.15	0.71	0.06
129.	Mucocellular papillary adenocarcinoma	–	Colon	M	51	1.01	0.09	1.18	0.08
130.	Large cell carcinoma	–	Lung	M	55	1.02	0.12	–	–
131.	Papillary adenocarcinoma	G2	Lung	F	61	1.03	0.01	0.82	0.07
132.	Papillary adenocarcinoma	–	Colon	F	67	1.08	0.14	–	–
133.	Small cell carcinoma	–	Lung	F	54	1.09	0.03	0.92	0.09
134.	Papillary adenocarcinoma	–	Kidney	M	64	1.09	0.02	–	–
135.	Papillary adenocarcinoma	–	Ovary	F	50	1.13	0.06	–	–
136.	Papillary adenocarcinoma	–	Ovary	F	55	1.18	0.17	–	–
137.	Papillary serous cystadenocarcinoma	G2	Ovary	F	52	1.23	0.03	–	–
138.	Adenocarcinoma	G3	Lung	F	54	1.24	0.07	–	–
139.	Planoepithelial carcinoma	G3	Larynx	M	64	1.31	0.13	–	–

The histological types and grades were estimated in routine pathological report.–: not estimated; G1: well differentiated; G2: moderately differentiated; G3: poorly differentiated.

**Figure 2:**
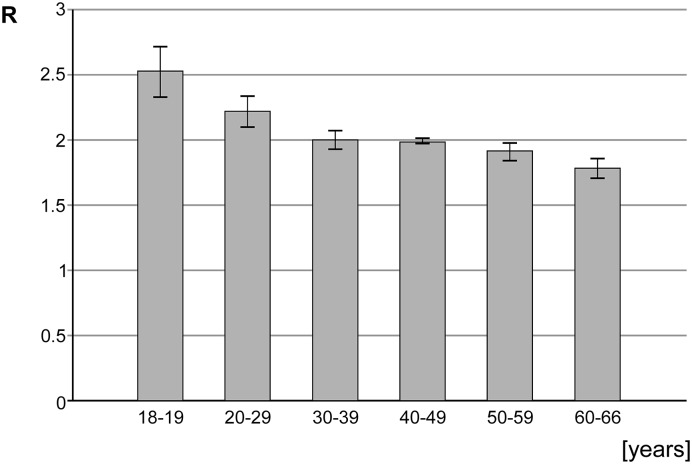
The m^5^C content (R) in DNA in the age subgroups of the cohort of healthy individuals

The control group consisted from 41 generally healthy persons, aged from 18 to 66 years (mean 43.1 ± 14.5 years), with 18 (43.9%) males and 7 (56.1%) females (Figure 2).

### Global DNA methylation in tumor tissue samples

**Figure 3:**
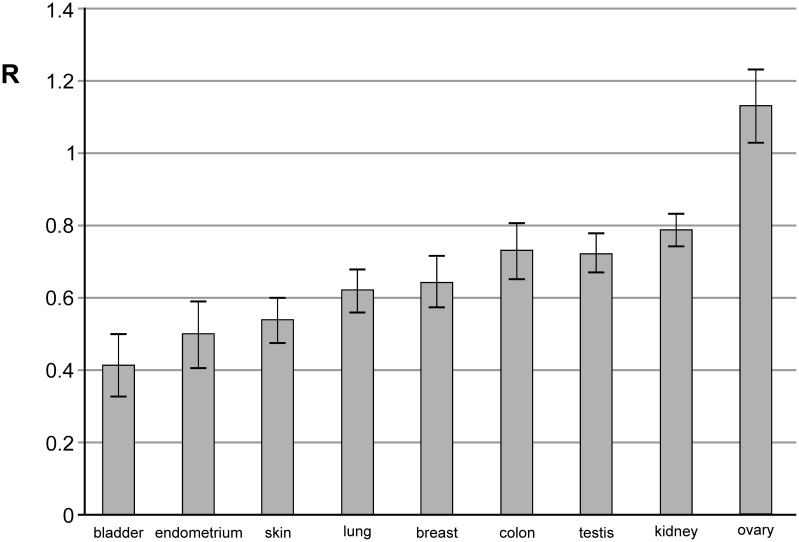
m^5^C content (R) in DNA isolated from different brain metastasis tissue in groups of the same site of origin The lowest values are presented in lung, skin, bladder, and endometrial cancer groups.

**Figure 4:**
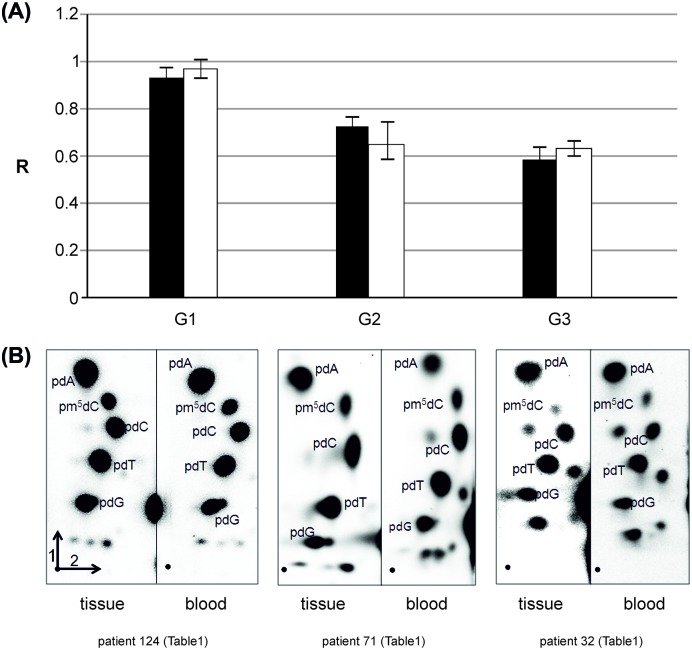
Comparison of m5C content in DNA of lung cancer brain metastasis and blood. (**A**) Amounts of m^5^C in DNA (R) in tumor tissue (white bars) and peripheral blood (black bars) of the same subjects with brain metastases from lung cancer. The one way ANOVA test comparing R for tissue and blood samples was performed for the whole group (*F*=0.115 and *P*=0.735), and for G2 (*F*=0.494 and *P*=0.492), and G3 (*F*=0.668 and *P*=0. 420) grade. G1 subgroup for lung cancer consisted, in the case of comparison with blood sample, of one patient. (**B**) Results of 2D TLC analysis of global methylation in DNA from tumor (left pictures) and peripheral blood (right pictures) of the same sample patients with brain metastases form lung cancer in different grades, from G1 to G3 and from left to right, respectively. The first and second separation dimension are labeled on chromatograms. [γ^32^P] 2’deoxynucleotides derived fom DNA hydrolysis are labeled. Unmarked spots show ribonucleotides, commonly found contamination with RNA (unpublished).

For all 139 patients the genomic level of m^5^C in DNA extracted from brain tumor tissue was analyzed (Table 1). The amount of m^5^C expressed as R coefficient (see Materials and methods) varies clearly between the patients (Table 1), and the groups divided by the site of origin (Figure 3). The differences in R between the groups of metastasis origin are statistically significant (*P*=0.0001). The level of m^5^C in DNA of brain metastatic tissue from the most abundant group of lung cancer negatively correlates with tumor grade. The well-differentiated (G1) tumors show higher m^5^C content than poorly differentiated (G3) and the difference is statistically significant (*P*=0.017) (Figure 4A). The lung cancer group was analyzed uniformly. The global DNA methylation in the subgroups of small cell lung cancer (SCLC) and NSCLC didn’t show statistically significant differences.

### Global DNA methylation in peripheral blood samples

**Figure 5:**
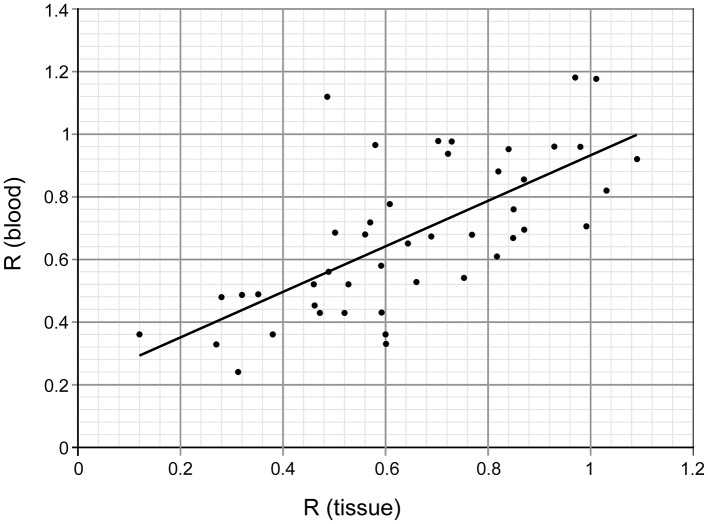
Comparison of genomic m^5^C contents in DNA from brain tumor tissue and peripheral blood of the same subjects Pearson’s *r* correlation factor is 0.69.

For 45 patients we also analyzed the genomic level of m^5^C in DNA from peripheral blood samples (Table 1). The direct comparison of the amount of m^5^C expressed as R coefficient (see Materials and methods) in tissue and blood for the same subjects showed very similar if not identical values (Table 1, Figures 4A and 5).

In the control group of generally healthy individuals the mean R coefficient was 2.07 ± 0.26, therefore significantly higher than in the brain metastases group. The ANOVA test for blood results in cancer patients and control group showed *F*=747.365 and *P*<0.001.

### Comparison of m^5^C content in DNA from tumor tissue and peripheral blood samples

m^5^C content in DNA from the brain metastasis tissue and peripheral blood of the same patient were almost identical. The one way ANOVA test values were: *F*=0.304156 and *P*=0.582686, showing no statistically significant differences between the groups. We performed a detailed analysis of the relationship between the groups analyzing mean values for tissue and blood global DNA methylation for lung cancer patients with different tumor grades (Figure 4A). The calculated r correlation coefficient for the whole group of patients with tissue–blood pair was 0.69 (Figure 5).

## Discussion

The assessment of epigenetic changes is one of the most promising in searching candidate markers for the early cancer detection. Since hematogenous dissemination of the tumor cells is the main mechanism for remote metastasis, a blood analysis may be a feasible approach for detecting systemic tumor cell spreading. Circulating tumor cells (CTCs) are considered to be the source of floating DNA, which is released into the circulation system upon the death of these cells. Tumor-related free-methylated DNA in blood of cancer patients has been assessed for their clinical utility.

A wide array of techniques are currently available to measure DNA methylation genome-wide and at the single gene level. However, such analysis requires large amounts of DNA, which makes it unsuitable for clinical analysis. At that background our method is a simple and sensitive way to detect global DNA methylation changes, that are correlated with cancer development and progression, and do not require large amounts of DNA, so it is also sufficient for blood analysis.

One can expect that the research efforts will focus on less invasively acquired material (blood, liquid biopsy, rather than surgical) analysis, and global (rather than a single-gene) DNA analysis, in order to comprehensively characterize the pathologic states. The factors leading to diseases act randomly and the general approach seems to be reflecting that.

The study of epigenetic alterations in the human genome has taken a central stage in an effort to better understand the molecular basis of human diseases beyond the well-documented realm of genetic events. The DNA methylation analysis at global and gene-specific levels has helped to shed light on gene function and has also uncovered a large number of genes whose expression is abolished, primarily thought as epigenetic mechanisms of disease [33–37]. Also, the fact that epigenetic changes are reversible, opens a new spectrum of potential treatment options, which may lead to the amelioration, or even elimination of the disease phenotype [38].

The most abundant group in our analysis was lung cancer patients, and for them we showed that decreasing methylation accompanies a higher tumor grade (Figure 4A). That observation is in concordance with our previous results for gliomas [30,31], highlights from genome-wide methylation studies [39] and already published observations that global demethylation results in higher cell homeostasis disturbance and leads to carcinogenesis [40–42].

The lung cancer group was analyzed without discrimination of SCLC and NSCLC subgroups. The reason for that was the histopathological distinction between SCLC and NSCLC is based on cytomorphological features and clinical characteristics. NSCLC is even defined as any type of epithelial lung cancer other than SCLC. NSCLC is composed of three cancer types, and is relatively insensitive to chemotherapy, compared with SCLC. SCLC metastasizes much faster than NSCLC and can be fatal in a few weeks if untreated, in contrast with most cases of NSCLC with metastases. However, it was shown that lung tumors can no longer be simply classified as NSCLC or SCLC, but histological subtyping and molecular testing are of paramount importance [43]. That molecular characterization includes not only genetic, but also epigenetic alterations [44]. Moreover, the histopathological transformation to small cell lung carcinoma in non-small cell lung carcinoma tumors was described proving that those types are not distinct [45]. That validates the joint consideration of lung cancer types in our research.

The lowest global DNA methylation values were observed in lung, skin, breast, bladder, and endometrial cancer groups. That correlates with clinical observations for melanoma, which is the most easily brain metastasizing tumor [8,46], and for lung cancer, where a large proportion of patients with NSCLC develop metastases during the course of the disease [47,48]. However, brain metastases from endometrial carcinoma, the most common gynecologic malignancy, are rare [49]. The number of cases for bladder cancer is even smaller [50], and brain metastasis development in those patients carries a very bad prognosis [51]. Therefore, we can presume that metastases to the brain happen in the most malignant of those tumors, what is reflected in low global DNA methylation or high demethylation.

We observed a suprisingly high DNA methylation in the group of metastases from ovarian carcinoma. That neoplasm is usually detected at the advanced stage and has a relatively poor prognosis [52]. Ovarian cancer metastasizes early in its development, often before it has been diagnosed. However, metastases to the brain are rare, even with the hematogenous route of spread [53]. That shows the presence of intravascular tumor cells in the cerebral circulation does not always lead to the development of brain metastases, which depends i.a. on the blood–brain barrier remodeling, host immune response, tissue neovascularization, the number of tumor emboli, and molecular tumor characteristics, e.g. expression of the proteins involved in cellular adhesion and migration [53–58]. Our patients were in the preliminary stage of the disease allowing for qualification to brain surgery. That can explain the higher DNA methylation level. However, to perform more accurate justification a more abundant patient group is required.

Blood is a very much desirable source of biomarkers mainly due to its ready accessibility. It seems that a discovery of protein markers in blood can be a daunting task because of a high dynamic range of protein concentrations, their heterogeneity and the multiple environmental factors (e.g. diet, stress, and disease). However, we decided to use of blood as a source of the epigenetic markers. We have already showed that global DNA methylation in peripheral blood samples reflects that of primary brain tumors’ tissue [31].

In our study the content of m^5^C in DNA from the brain tumor tissue and peripheral blood of the same metastatic brain tumor patient is almost identical (Figure 5). The relations between them were checked with ANOVA test (*P*-value 0.58, *F*-test 3.95). The correlation analysis result was 0.69, what is regarded as a quite strong correlation. Therefore it seems that R in DNA from peripheral blood can be used as a diagnostic tool in neurooncology. While analyzing only the level of m^5^C in DNA of brain tumor tissue and blood of the same patient in lung cancer group, we observed that it negatively correlates with the tumor grade. The results of statistical comparison between R values for tissue and blood within the grade groups also showed no significant differences (Figure 4A).

One can ask whether genomic methylation level can be a good diagnostic marker for the early tumor detection in the clinical practice [59]. We have recently found that the extent of DNA demethylation process is different and specific for various diseases [31]. The lowest level of DNA methylation correlates well with aggressive tumors like glioblastoma and anaplastic astrocytoma. Because there are differences in m^5^C content in DNA from blood of patients with other diseases, one can use the global methylation as a diagnostic tool [31,60,61].

The most important question is why and how the global DNA methylation could be the same in tumor tissue and blood samples. The first possible answer is based on the mechanism in which DNA methylation is affected by ROS. Because it’s a random and global process not limited to the certain area or cell type, the disease signs can be found in the whole body, not only in the foci where tumors are localized [62,63]. The other explanation is the presence of CTCs and circulating tumor DNA in peripheral blood at higher levels than that of healthy individuals, which was already shown in many cancers [64,65]. These carry the tumor methylation profile and have an impact on the detected level of global DNA methylation.

The present paper shows that the global hypomethylation of DNA from metastatic brain tumors differs depending on the site of origin and tumor malignancy. The level of methylation values for tissue and peripheral blood samples from patients with metastatic brain tumors are almost on the same level. That stresses to an extent of the neoplasm, but also enables to use peripheral blood as a sample for describing the metastatic disease and brain metastases in particular. We showed that DNA methylation level (R value) in tumor tissue and peripheral blood can be used as a diagnostic marker for brain metastases.

Given the role of aberrant DNA methylation in cancer initiation and progression, distinct effort has been put towards the development of strategies which could facilitate early cancer detection. It is now clear that aberrant DNA methylation is an early event in tumor development, as indicated by reports where aberrantly hypermethylated sites could be detected in seemingly normal epithelia from patient’s years before the overt development of cancer [66]. Thus, utilizing DNA methylation as a biomarker might prove to be a useful tool not only for early diagnosis, but also for the detection and assessment of high-risk individuals.

## Clinical perspectives

- Epigenetics plays a crucial role in cancer development and progression. ROS react and damage the epigenetic mark, m^5^C.
- The global DNA m^5^C in brain metastases changes in the broad range. Global DNA methylation in tumor tissues and peripheral blood overlap.
- The genomic DNA methylation is a useful marker for brain metastases diagnosis.

## Abbreviations

CNS: central nervous system
DNA: deoxyribonucleic acid
i.a.: inter alia
KPS: Karnofsky Performance Score
m^5^C: 5-methylcytosine
NSCLC: non-small cell lung cancer
OS: overall survival
ROS: reactive oxygen species
SCLC: small cell lung cancer
TLC: thin layer chromatography
